# Long-Term Outcomes of Concurrent Chemoradiotherapy With S-1 in Older Patients With Esophageal Cancer: A Secondary Analysis of a Randomized Clinical Trial

**DOI:** 10.1001/jamanetworkopen.2026.3541

**Published:** 2026-03-27

**Authors:** Yongling Ji, Min Fang, Weiguo Zhu, Yanguang Yang, Jun Ma, Li Zhang, Jiancheng Li, Hua Tao, Jianhong Xia, Haihua Yang, Jin Huang, Yong Bao, Dexi Du, Degan Liu, Xiusheng Wang, Chaoming Li, Xinmei Yang, Ming Zeng, Zhigang Liu, Wen Zheng, Juan Pu, Jun Chen, Wangyuan Hu, Xinyi Wang, Peijing Li, Jin Wang, Yujin Xu, Xiao Zheng, Keying Chen, Wanwei Wang, Guangzhou Tao, Jing Cai, Jizhong Zhao, Jun Zhu, Ming Jiang, Yan Yan, Guoping Xu, Wenjing Xu, Shanshan Bu, Binbin Song, Ke Xie, Shan Huang, Yuanda Zheng, Liming Sheng, Xiaojing Lai, Ying Chen, Lei Cheng, Xiao Hu, Wenhao Ji, Yue Kong, Xiaofu Yu, Huizhang Li, Runhua Li, Rong Huang, Han He, Xianghui Du, Ming Chen

**Affiliations:** 1 Zhejiang Cancer Hospital, Hangzhou Institute of Medicine (HIM), Chinese Academy of Science, Hangzhou, China; 2 Sun Yat-sen University Cancer Center, State Key Laboratory of Oncology in South China, Guangdong Provincial Clinical Research Center for Cancer, United Laboratory of Frontier Radiotherapy Technology of Sun Yat-sen University & Chinese Academy of Sciences Ion Medical Technology Co Ltd, Guangzhou, China; 3 Department of Radiation Oncology, The Affiliated Huaian No.1 People's Hospital of Nanjing Medical University, Huaian, China; 4 Department of Radiation Oncology, Nantong Tumor Hospital, Nantong, China; 5 Department of Radiation Oncology, First Affiliated Hospital of University of Science and Technology of China, Hefei, China; 6 Department of Oncology, Chongqing Sanxia Central Hospital, Chongqing, China; 7 Department of Thoracic Radiation, Fujian Provincial Cancer Hospital, Fuzhou, China; 8 Department of Radiation Oncology, Jiangsu Cancer Hospital, Nanjing, China; 9 Department of Radiation Oncology, Huaian Second People's Hospital, Huaian, China; 10 Department of Radiation Oncology, Taizhou Hospital of Zhejiang Province, Taizhou, China; 11 Department of Radiation Oncology, The First People's Hospital of Changzhou, Changzhou, China; 12 Department of Radiation Oncology, The First Affiliated Hospital of Sun Yat-sen University, Guangzhou, China; 13 Department of Radiation Oncology, Lishui Municipal Central Hospital, Lishui, China; 14 Department of Oncology, Xinghua City People's Hospital, Xinghua, China; 15 Department of Radiation Oncology, Henan Cancer Hospital, Zhengzhou, China; 16 Department of Oncology, Maoming People's Hospital, Maoming, China; 17 Department of Radiation Oncology, The First Hospital of Jiaxing, Jiaxing, China; 18 Sichuan Provincial People's Hospital, University of Electronic Science and Technology of China, Chengdu, China; 19 The Tenth Affiliated Hospital of Southern Medical University, Dongguan People's Hospital, Dongguan, China; 20 Department of Oncology, Shangrao People's Hospital, Shangrao, China; 21 Department of Oncology, Lianshui County People's Hospital, Lianshui, China; 22 Department of Chemoradiotherapy, Yinzhou People's Hospital, Ningbo, China; 23 Department of Radiation Oncology, Jinhua People’s Hospital, Jinhua, China; 24 Department of Population Health Sciences, Duke University School of Medicine, Durham, North Carolina; 25 Department of Radiation Oncology, The First People's Hospital of Foshan, Foshan, China

## Abstract

**Importance:**

Most older patients with esophageal cancer (EC) are unable to complete standard platinum-based concurrent chemoradiotherapy (CCRT) due to reduced organ reserve, comorbidities, and malnutrition. A new treatment option—CCRT with S-1—has been found to have high efficacy and fewer toxic effects for this population, yet long-term data supporting its use remain limited.

**Objective:**

To evaluate the long-term outcomes of CCRT with S-1 vs radiotherapy (RT) alone in older patients with EC.

**Design, Setting, and Participants:**

This secondary analysis of a phase 3 randomized clinical trial conducted at 23 centers in China was not prespecified in the trial protocol. Patients aged 70 to 85 years with histologically confirmed EC were enrolled between June 1, 2016, and August 31, 2018. Data cutoff date was February 1, 2025, with an additional follow-up of 54 months beyond the primary analysis. Data were analyzed from February 1 to April 1, 2025.

**Interventions:**

Patients were randomly assigned 1:1 to receive CCRT with S-1 consisting of 54 Gy in 30 fractions with S-1, 70 mg/m^2^ per day on days 1 to 14 and 29 to 42, or RT alone consisting of 60 Gy in 30 fractions, 2.0 Gy per day 5 days per week.

**Main Outcomes and Measures:**

The primary outcome was overall survival (OS). Secondary outcomes were progression-free survival (PFS), cause-specific mortality, cumulative incidence of death from EC or other reasons, and cumulative incidences of locoregional or distant metastasis during treatment or relapse after treatment.

**Results:**

A total of 298 patients (median [IQR] age, 77 [74-79] years; 180 males [60.4%]) were enrolled. There were 151 patients (50.7%) clinically diagnosed with stage III to IV disease. With a median (IQR) follow-up of 87 (85-92) months, the median OS was 24.7 (95% CI, 21.2-37.6) months in the CCRT group and 15.1 (95% CI, 12.4-18.6) months in the RT group (hazard ratio [HR], 0.69; 95% CI, 0.53-0.90; *P* = .005). The 5-year OS rates were 33.5% (95% CI, 26.7%-42.1%) and 24.4% (95% CI, 18.3%-32.4%) for the CCRT and RT groups, respectively; the 8-year OS rates were 26.2% and 16.1%, respectively. The median PFS was 18.7 (95% CI, 13.1-25.8) months in the CCRT group and 9.2 (95% CI, 7.9-12.7) months in the RT group (HR, 0.69; 95% CI, 0.54-0.90; *P* = .005). Cause-specific analyses showed reduced EC-related mortality with CCRT (HR, 0.67; 95% CI, 0.50-0.89; *P* = .005), with 8-year absolute risks of 58.5% vs 72.9%, respectively, and no excess noncancer-related mortality.

**Conclusions and Relevance:**

In this secondary analysis of a randomized clinical trial, long-term results showed that CCRT with S-1 was associated with a sustained survival benefit compared with RT alone, without increased noncancer-related mortality. This finding supports CCRT with S-1 as the preferred regimen for patients aged 70 to 85 years with EC.

**Trial Registration:**

ClinicalTrials.gov Identifier: NCT02813967

## Key Points

**Question:**

Is concurrent chemoradiotherapy (CCRT) with S-1 associated with differences in survival compared with radiotherapy alone in older patients with esophageal cancer (EC)?

**Findings:**

In this secondary analysis of a randomized clinical trial involving 298 patients with EC, the first prospective long-term data (median follow-up of 87 months) on treatment outcomes were reported. Patients in the CCRT with S-1 group showed significantly better overall survival than those in the RT-alone group, and long-term follow-up revealed no increase in noncancer-related mortality among patients receiving CCRT with S-1.

**Meaning:**

These results support CCRT with S-1 as an effective and tolerable treatment option for older patients with EC, addressing the critical evidence gap for this underrepresented population.

## Teaser

This secondary analysis of a randomized clinical trial reports the long-term survival rates and cancer- and noncancer-related causes of death associated with concurrent chemoradiotherapy with S-1 in older patients in China.

## Introduction

Esophageal cancer (EC) ranks as the seventh leading cause of cancer-related mortality worldwide, with approximately 40% of cases diagnosed in patients 70 years or older.^1,2,3^ The incidence of EC increases substantially with age, and with global aging and increasing life expectancy, the number of older patients with EC is projected to rise substantially.^4^ Although concurrent chemoradiotherapy (CCRT) with platinum-based doublet chemotherapy is the standard treatment for inoperable, locally advanced EC,^5,6^ most older patients do not tolerate this regimen because of a decline in organ function, frequent comorbidities, and malnutrition.^7,8,9,10,11^ Optimized treatment specifically for older patients has been investigated in phase 1, 2, and 3 randomized clinical trials of CCRT with oral fluoropyrimidine S-1 (tegafur, 5-chloro-2,4-dihydroxypyridine, and potassium oxonate) in patients with EC aged 70 years or older.^12,13,14^ In the primary analysis of our multicenter phase 3 trial (median follow-up, 33.9 months), CCRT with S-1 demonstrated superior efficacy to radiotherapy (RT) alone, achieving a 2-year overall survival (OS) rate of 53.2% vs 35.8% (hazard ratio [HR], 0.63; 95% CI, 0.47-0.85; *P* = .002).^14^ The regimen was well tolerated, with grade 3 or higher adverse events (AEs) occurring in less than 10% of patients. Subsequent prospective studies confirmed the tolerability and short-term efficacy of CCRT with S-1.^15^

However, early survival gains do not necessarily translate into durable benefit for older patients. Over time, competing noncancer-related mortality and treatment-related toxic effects can accumulate and erode long-term outcomes. In some cohorts, deaths from nontumor causes exceed EC-specific mortality beyond 5 years after definitive chemoradiotherapy.^16^ Yet long-term data specific to older adults remain scarce. To address this gap, we conducted the present study to evaluate the long-term outcomes of CCRT with S-1 vs RT alone in older patients with EC using the 7-year follow-up results of our multicenter phase 3 trial. This study offers further insight into an optimized treatment strategy for older patients with EC.

## Methods

### Study Design and Participants

This secondary analysis of an open-label, phase 3 randomized clinical trial, which enrolled participants at 23 centers in China between June 1, 2016, and August 31, 2018, was not prespecified in the trial protocol. The Zhejiang Cancer Hospital Ethics Committee approved the trial protocol and related documents (Supplement 1). Written informed consent was obtained from all participants. We followed the Consolidated Standards of Reporting Trials (CONSORT) reporting guideline.

Details of the trial design have been reported previously.^14^ Briefly, patients aged 70 to 85 years with EC, with an Eastern Cooperative Oncology Group performance status of 0 (indicating fully active and able to carry on all predisease activities without restriction) or 1 (indicating restricted in physically strenuous activity but ambulatory and able to carry out light work), and with histologically confirmed stage IB to IVB disease (using American Joint Committee on Cancer *Cancer Staging Manual,* sixth edition; stage IVB included only supraclavicular or celiac lymph node metastasis and no other distant metastasis) were randomized. Patients were excluded if they had a tracheoesophageal fistula, active infection, interstitial pneumonia, severe cardiovascular disease, malignant pleural effusion, pericardial effusion, or other concomitant cancers.

Patients were randomly assigned 1:1 to the CCRT with S-1 group or the RT-alone group using a stratified permuted-block method. Random assignments were stratified according to age (<80 vs ≥80 years) and tumor length (<5 vs ≥5 cm) by a statistician (R.L.) at Zhejiang Cancer Hospital randomization center. Group assignments were provided to the investigators via telephone. Patients and physicians were not masked to the group assignments.

### Interventions

Eligible patients in the CCRT group were treated with RT (54 Gy in 30 fractions, 1.8 Gy per day 5 days per week) with concurrent S-1 administration (70 mg/m^2^ per day orally on days 1-14 and 29-42), supplied by Shandong New Time Pharmaceutical LTD. Patients assigned to the RT group were treated with RT alone (60 Gy in 30 fractions, 2.0 Gy per day 5 days per week).

All patients were radiated by the 3-dimensional conformal RT or intensity-modulated RT using a linear accelerator with 6-MV to 10-MV photons. The gross tumor volume was defined as the primary tumor, and the involved lymph nodes were determined using all available information (physical examination, endoscopy, ultrasonography endoscopy, neck-thorax-abdomen computed tomography, and positron emission computed tomography). The clinical tumor volume was defined as the primary tumor plus 3-cm superior and inferior expansion margins and a 1-cm radial expansion margin. The planning target volume was defined as a 0.5-cm to 1-cm margin around the clinical tumor volume to account for tumor motion and setup variations. The follow-up schedule and imaging frequency were followed in accordance with the study protocol, every 3 months during the first 2 years, and every 6 months thereafter. To ensure consistency and adherence to the protocol, regular investigator meetings were held to review trial progress, discuss protocol-related issues, and address any deviations or operational challenges in a timely manner.

### Outcomes

The primary outcome was OS, which was calculated from the date of randomization to date of death from any cause or last follow-up. The secondary outcomes were progression-free survival (PFS), cause-specific mortality, cumulative incidence of death from EC or other reasons, and cumulative incidences of locoregional or distant metastasis during treatment or relapse after treatment. PFS was defined as the time from randomization to disease progression or death. Cause-specific mortality was defined as death from EC due to locoregional recurrence, distant metastasis during treatment, or relapse after treatment. Locoregional recurrence was defined as the first documented event of tumor progression at the primary site, mediastinum, or supraclavicular region. Distant metastasis was defined as the first documented event of tumor progression in lymph nodes higher than the supraclavicular region or lower than the celiac lymph nodes, peritoneal carcinomatosis, malignant pleural effusions, or hematogenous metastases. Since we sought to analyze the effect of CCRT with S-1 on the risk of death from causes other than EC, treatment-related deaths were not classified as deaths from EC. For cumulative incidence of death from EC, death from other causes precluded the event of interest and was defined as competing risk.

### Statistical Analysis

Analysis (data cutoff date: February 1, 2025) was based on the intent-to-treat population. Survival analysis was performed using the Kaplan-Meier method and compared using the log-rank test. For a more clinically meaningful comparison, we compared the restricted mean survival time (RMST). Univariate and multivariable Cox proportional hazards regression models were used to determine factors associated with OS. The Pearson χ^2^ test or Fisher exact test was used to compare categorical variables between groups, while the Wilcoxon rank sum test was applied to compare continuous variables.

The effect of CCRT or RT on EC-related or other-cause mortality and on locoregional recurrence and/or distant metastasis was assessed using Fine and Gray competing risk models, with death from an unknown cause considered as other-cause mortality. Differences were considered statistically significant at 2-sided *P* < .05. All analyses were performed using R version 3.6.1 (R Core Team). Data were analyzed from February 1 to April 1, 2025.

## Results

### Patients and Treatment

**Figure 1.**
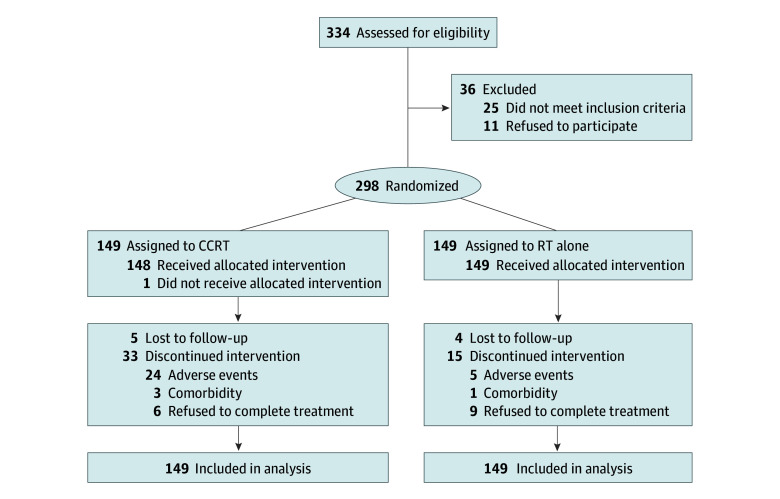
Participant Flow Diagram Study data were collected up to the cutoff date (February 1, 2025). CCRT indicates concurrent chemoradiotherapy; RT, radiotherapy.

The trial enrolled 298 patients with EC who were randomly assigned to receive either CCRT with S-1 (n = 149) or RT alone (n = 149) (Figure 1). The baseline patient and disease characteristics are reported in the Table. Among these patients, the median (IQR) age was 77 (74-79) years in the CCRT group and 77 (74-80) years in the RT group, with 39 patients (26.2%) in the CCRT group and 37 patients (24.8%) in the RT group being 80 years or older. Overall, there were 180 male (60.4%) and 118 female (39.6%) patients, of whom 151 (50.7%) had stage III or IV disease and 296 (99.3%) had squamous cell carcinoma.

**Table.** Baseline Characteristics of the Intention-to-Treat Population

Characteristics	Patients, No. (%)	
	CCRT group (n = 149)	RT group (n = 149)
Age, y		
Median (IQR)	77 (74-79)	77 (74-80)
70-79	112 (75.2)	110 (73.8)
≥80	37 (24.8)	39 (26.2)
Sex		
Male	89 (59.7)	91 (61.1)
Female	60 (40.3)	58 (38.9)
ECOG performance status^a^		
0	22 (14.8)	16 (10.7)
1	127 (85.2)	133 (89.3)
BMI		
Median (IQR)	21.5 (19.5-23.8)	21.5 (19.0-23.4)
<18.5	27 (18.1)	32 (21.5)
≥18.5	122 (81.9)	117 (78.5)
NRS-2002 score^b^		
<3	90 (60.4)	82 (55.0)
≥3	59 (39.6)	67 (45.0)
CCI^c^		
0	106 (71.1)	96 (64.4)
≥1	43 (28.9)	53 (35.6)
Histological type		
SCC	148 (99.3)	148 (99.3)
Adenocarcinoma	1 (0.7)	1 (0.7)
Tumor length, cm		
<5	59 (39.6)	61 (40.9)
≥5	90 (60.4)	88 (59.1)
Tumor location		
Cervical	8 (5.4)	5 (3.4)
Upper thoracic	47 (31.5)	46 (30.9)
Middle thoracic	80 (53.7)	83 (55.7)
Lower thoracic	14 (9.4)	15 (10.1)
Cancer stage (AJCC sixth edition)		
IIA	48 (32.2)	62 (41.6)
IIB	21 (14.1)	16 (10.7)
III	71 (47.7)	62 (41.6)
IVA	5 (3.4)	3 (2.0)
IVB	4 (2.7)	6 (4.0)

Abbreviations: AJCC, American Joint Committee on Cancer *Cancer Staging Manual*; BMI, body mass index (calculated as weight in kilograms divided by height in meters squared); CCI, Charlson Comorbidity Index; CCRT, concurrent chemoradiotherapy; ECOG, Eastern Cooperative Oncology Group; NRS-2002, Nutritional Risk Screening 2002; RT, radiotherapy; SCC, squamous cell carcinoma.

^a^ ECOG performance status: 0 (indicating fully active and able to carry on all predisease activities without restriction) or 1 (indicating restricted in physically strenuous activity but ambulatory and able to carry out light work).

^b^ NRS-2002 score range: 0 to 7, with the highest score indicating severe nutritional risk.

^c^ CCI range: 0 to 37, with the highest score indicating multiple comorbidities and a poorer prognosis.

The treatment adherence and safety profiles have been described previously.^14^ In brief, 133 patients (89.3%) completed the prescribed RT and 115 patients (77.2%) completed oral chemotherapy with S-1 in the CCRT group. In the RT group, 134 patients (89.9%) completed the prescribed RT. Treatment-related deaths were observed in 3 patients (2.0%) in the CCRT group and 4 patients (2.7%) in the RT group.

### Survival Analysis

**Figure 2.**
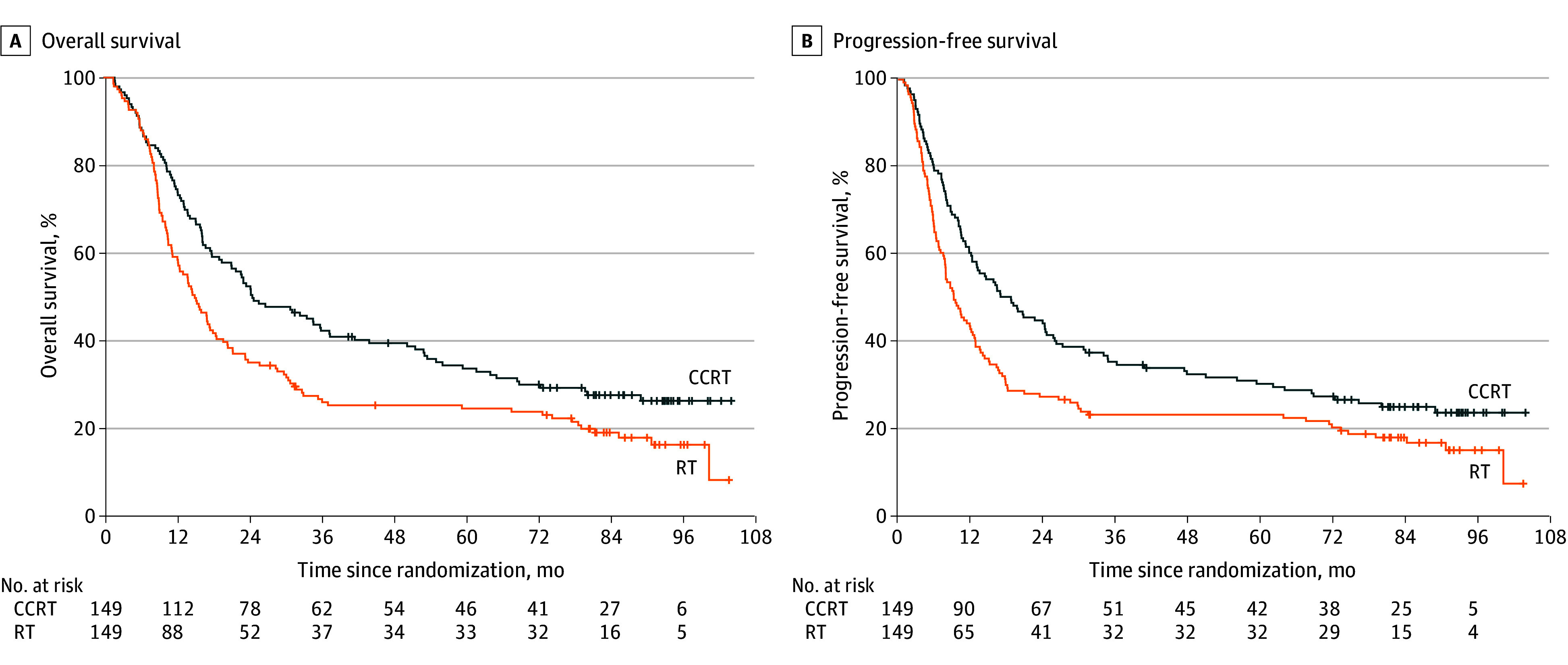
Kaplan-Meier Analysis of Overall Survival and Progression-Free Survival CCRT indicates concurrent chemoradiotherapy; RT, radiotherapy.

The median (IQR) follow-up was 89.5 (81.7-94.2) months in the CCRT group and 86.5 (81.6-93.2) months in the RT group. A total of 229 OS events occurred: 107 in the CCRT group and 122 in the RT group. The median OS was 24.7 (95% CI, 21.2-37.6) months in the CCRT group and 15.1 (95% CI, 12.4-18.6) months in the RT group, with an HR of 0.69 (95% CI, 0.53-0.90; *P* = .005). OS rates in the CCRT and RT groups were 33.5% (95% CI, 26.7%-42.1%) and 24.4% (95% CI, 18.3%-32.4%) at 5 years and 26.2% (95% CI, 19.7%-34.8%) and 16.1% (95% CI, 0.7%-24.3%) at 8 years (Figure 2). The RMST of OS was 33.8 (95% CI, 28.0-39.7) months in the RT group and 45.5 (95% CI, 39.2-51.9) months in the CCRT group (*P* = .008) (eFigure 1 in Supplement 2).

At the time of analysis, 91 patients (61.1%) in the CCRT group and 107 patients (71.8%) in the RT group had disease progression. The median PFS was 18.7 (95% CI, 13.1-25.8) months in the CCRT group and 9.2 (95% CI, 7.9-12.7) months in the RT group (HR, 0.69; 95% CI, 0.54-0.90; *P* = .005). PFS rates in the CCRT and RT groups were 30.5% (95% CI, 23.9%-38.9%) and 23.4% (95% CI, 17.5%-31.3%) at 5 years and 23.9% (95% CI, 17.6%-32.3%) and 15.3% (95% CI, 10.0%-23.4%) at 8 years (Figure 2). The RMST of PFS was 28.8 (95% CI, 22.9-34.8) months in the RT group and 39.8 (95% CI, 33.3-46.3) months in the CCRT group (*P* = .02) (eFigure 1 in Supplement 2).

The updated OS for the patient subgroups was consistent with previous reports (eFigure 2 in Supplement 2). OS benefited the CCRT group vs the RT group across all subgroups, except for patients with a lower thoracic tumor location (HR, 1.13; 95% CI, 0.49-2.62). For the survival of patients older than 80 years, CCRT also could improve survival. eTable 1 in Supplement 2 presents the multivariable analysis of OS. The following variables were associated with improved OS: CCRT (HR, 0.68; 95% CI, 0.52-0.88), age 70 to 79 years (HR, 1.44; 95% CI, 1.08-1.92), female sex (HR, 1.38; 95% CI, 1.05-1.80), Charlson Comorbidity Index of 0 (HR, 1.34; 95% CI, 1.00-1.80), and stage II disease (HR, 1.33; 95% CI, 1.01-1.75).

### Cause-Specific Mortality

Of the 149 patients in the CCRT group, 86 (57.7%) died of EC and 17 (11.4%) died from other causes. Of the 149 patients in the RT group, 105 (70.5%) died of EC and 15 (10.1%) died of other causes. An additional 4 patients in the CCRT group and 2 in the RT group died of unknown causes. The causes of death are specified in eTable 2 of Supplement 2. With an HR of 0.64 (95% CI, 0.48-0.85; *P* = .002), the median cancer-specific OS was 33.7 (95% CI, 23.7-53.7) months in the CCRT group and 17.1 (95% CI, 14.2-21.5) months in the RT group (eFigure 3 in Supplement 2).

**Figure 3.**
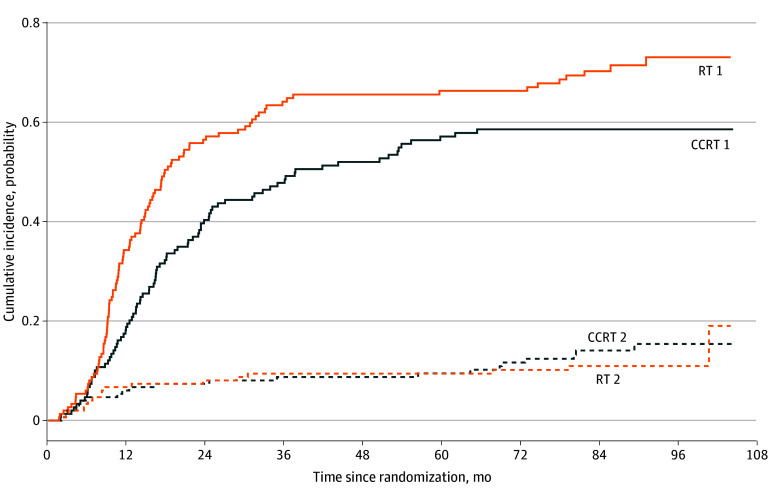
Line Graph of Competing Risk Analysis for Cumulative Incidence of Death by Cause CCRT indicates concurrent chemoradiotherapy; RT, radiotherapy; solid lines, death by esophageal cancer; and dashed lines, death by other cause.

Patients in the CCRT group were at a lower risk of dying from EC than patients in the RT group (HR, 0.67; 95% CI, 0.50-0.89; *P* = .005), with 8-year absolute risks of 58.5% (95% CI, 50.0%-66.0%) vs 72.9% (95% CI, 64.1%-79.9%). Death from other causes was comparable between the CCRT and RT groups (HR, 1.11; 95% CI, 0.81-1.53; *P* = .51), with 8-year absolute risks of 15.4% (95% CI, 9.8%-22.2%) vs 11.0% (95% CI, 6.5%-16.7%) (Figure 3).

### Recurrence

In the CCRT group, 48 of 149 patients (32.2%) had isolated locoregional recurrence compared with 70 of 149 patients (47.0%) in the RT group (HR, 0.52; 95% CI, 0.36-0.75; *P* < .001). Within 3 years of follow-up, 44 of 48 patients (91.7%) in the CCRT group and 63 of 70 patients (90.0%) in the RT group developed locoregional recurrence.

**Figure 4.**
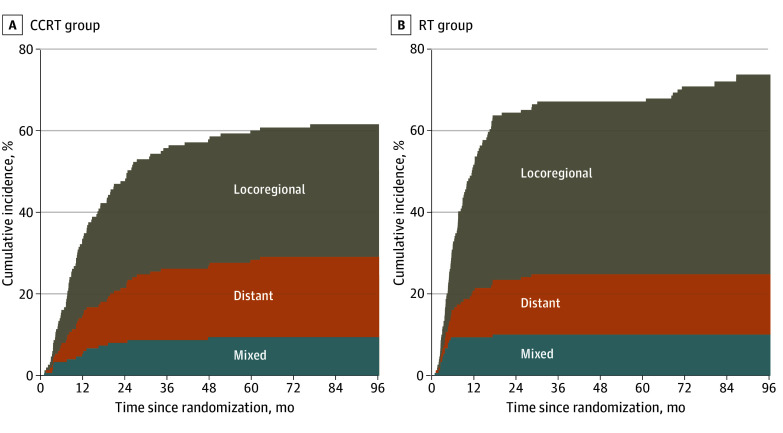
Line Graphs of Cumulative Incidence of Recurrence Location With the Concurrent Chemoradiotherapy (CCRT) and Radiotherapy (RT) Group Shaded areas represent mutually exclusive recurrence patterns: gray, isolated locoregional recurrence; orange, isolated distant metastasis; and blue, synchronous locoregional recurrence and distant metastasis.

Isolated distant metastasis developed in 29 of the 149 patients (19.5%) in the CCRT group and in 22 of the 149 patients (14.8%) in the RT group (HR, 1.00; 95% CI, 0.57-1.73; *P* = .99). Synchronous locoregional recurrence and distant metastasis developed in 14 of 149 patients (9.4%) in the CCRT group and in 15 of 149 patients (10.1%) in the RT group (HR, 0.78; 95% CI, 0.38-1.63; *P* = .51). The incidences of isolated distant metastasis and synchronous locoregional recurrence and distant metastasis were comparable in both groups (eTable 3 in Supplement 2). Cumulative incidence of recurrence is shown in Figure 4.

## Discussion

Long-term efficacy is essential in developing clinical treatment strategies, especially for older patients who are more susceptible to late treatment–related toxic effects that may compromise survival outcomes. However, comprehensive data regarding long-term outcomes in older patients with EC remain limited. To our knowledge, this secondary analysis was the first prospective study to report long-term follow-up results in older patients with EC. With a median follow-up of more than 7 years, the multicenter, phase 3 randomized clinical trial demonstrated significant survival benefits of CCRT with S-1 compared with RT alone (HR, 0.69; 95% CI, 0.53-0.90). CCRT achieved a 5-year survival rate of 33.5% in the CCRT group, surpassing historical benchmarks from retrospective analyses that documented 5-year survival rates of 17.8% to 21.4% for platinum-based CCRT in comparable cohorts.^9,17^ This finding aligns with a US National Cancer Database analysis reporting that the 5-year survival rate was less than 20% of patients 70 years or older with stage II to III EC undergoing CCRT.^18^ Subgroup analyses in the present study suggested that survival benefits were more favorable for CCRT across most subgroups, including patients 80 years or older and those with comorbidities or malnutrition, who are traditionally considered high risk for toxic effects. These findings demonstrate that CCRT with S-1 may offer sustained survival benefits for a broad population of older patients with EC, supporting its potential as a preferred treatment option for this group.

Treatment toxic effect is another critical consideration when making treatment decisions for the geriatric oncology population.^19^ Contemporary evidence indicates that older individuals experience substantially increased toxic effects from standard CCRT with platinum-based chemotherapy, manifested by 13% to 18% treatment-related mortality rates and low treatment completion rates of only 9% to 38.5%.^9,10,11,19^ In contrast, our trial found that 77.2% of the patients in the CCRT group completed the planned treatment, with a less than 10% incidence of grade 3 or higher AEs and a treatment-related mortality rate of 2%.^14^ Similar findings were reported in other prospective studies.^15,20,21^ For example, a phase 3 randomized clinical trial by Wang et al^15^ reported a 72.8% completion rate for S-1–based CCRT in patients 70 years or older with esophageal squamous cell carcinoma, with grade 3 or higher toxic effects (other than esophagitis [11.9%]) occurring in less than 10% of patients and a treatment-related mortality rate of 2.8%. The CCRT group did show more frequent AEs, especially hematologic toxic effects, and higher rates of treatment discontinuation than the RT-alone group. However, CCRT with S-1 showed better tolerance and less toxic effects than standard platinum-based CCRT. Our extended follow-up further confirmed the long-term safety of this regimen, with no excess noncancer-related mortality in the CCRT group compared with the RT-alone group.

The RT-alone group achieved a 5-year OS rate of 24.4% despite approximately 50% of patients having stage III to IV disease, which starkly contrasts with the historical data in the Radiation Therapy Oncology Group 85-01 trial, which reported a 3-year OS rate of 0 for adults with EC who received RT alone.^22^ This discrepancy in outcomes may be partly attributed to differences in histological subtypes, advances in radiotherapy techniques, and improvements in supportive care. Age-related tumor biological divergence may also play a role. A large-scale SEER (Surveillance, Epidemiology, and End Results Program) database analysis revealed that older patients with non–small cell lung cancer had substantially lower risks of lymph node and distant metastases than younger patients.^23^ Other studies have also identified differences in tumor biological process, including histological subtype, molecular markers, and treatment responses, between older and younger patients.^24,25,26^ These findings underscore the need for specific therapeutic strategies in older patients.^27^

Our analysis showed that CCRT significantly reduced the locoregional recurrence rate, while maintaining comparable distant metastasis rates. This finding highlights the need for more effective systemic therapies. The combination of CCRT with immunotherapy is a promising strategy. Immunotherapy has already shown significant survival benefits in metastatic EC and in patients who do not achieve a pathological complete response following neoadjuvant chemoradiotherapy.^28,29^ Several phase 3 randomized clinical trials evaluating chemoradiotherapy plus immunotherapy for unresectable locally advanced EC are ongoing.^30,31,32,33^ However, this combination is likely to increase toxic effects, and older patients may not tolerate standard platinum-based CCRT with immunotherapy. Given the favorable efficacy and low toxic effect profile of CCRT with S-1, this regimen may offer a more suitable backbone for integrating immunotherapy in older patients, warranting further investigation.

### Limitations

This study has several limitations. First, the study population consisted predominantly of patients with esophageal squamous cell carcinoma, with only 2 adenocarcinoma cases enrolled. This sample reflected the epidemiological pattern of EC in China, where esophageal squamous cell carcinoma was the dominant subtype. Therefore, our findings may not be generalizable to patients with adenocarcinomas. Second, the treatment after disease progression may not have been recorded accurately during follow-up. During the COVID-19 pandemic, certain health care resources were redirected and some patients faced difficulties in attending follow-up visits as scheduled, which may have potential impacts on survival outcomes. In addition, a proportion of patients were followed up by telephone after primary analysis and late AEs may not have been comprehensively assessed or consistently graded, which could have resulted in underreporting. Third, comprehensive geriatric assessments were not conducted, despite their growing recognition as a valuable tool for evaluating functional status, comorbidities, and overall health of older patients, which can help guide treatment decisions and estimate outcomes.^7^

## Conclusions

In this secondary analysis of a phase 3 randomized clinical trial of CCRT with S-1 in older patients with EC, these long-term findings supported CCRT with S-1 as a tolerable and effective alternative for older patients, demonstrating sustained survival benefits compared with RT alone. Given its favorable tolerability and safety profile, the integration of immunotherapy with CCRT with S-1 merits further investigation.

## Notes

Supplement 1.
Trial Protocol

Supplement 2. **eTable 1.** Overall Survival and Progression-Free Survival in 298 Patients: Multivariate Cox Proportional Analysis **eTable 2.** All Causes of Death Recorded Until the Final Day of Follow-up **eTable 3.** Site of First Progression **eFigure 1.** Restricted Mean Survival Time **eFigure 2.** Subgroup Analyses of Overall Survival in the Intention-to-Treat Population **eFigure 3.** Cancer-Specific Survival

Supplement 3.
Data Sharing Statement
