# Supplementary material for: Long-Term Outcomes of Concurrent Chemoradiotherapy With S-1 in Older Patients With Esophageal Cancer: A Secondary Analysis of a Randomized Clinical Trial
Source: JAMA Netw Open. 2026 Mar 27;9(3):e263541. doi: 10.1001/jamanetworkopen.2026.3541 (PMC13032157; doi:10.1001/jamanetworkopen.2026.3541)
Supplement: Supplement 1. — Trial Protocol [file jamanetwopen-e263541-s001.pdf]

Concurrent Chemoradiotherapy with S-1 versus radiotherapy alone in elderly patients with esophageal cancer: a multicenter, randomized, phase III trial

(June 7, 2016，Version 2.0)

Principal investigators

Professor Ming Chen

Department of Radiation Oncology, Zhejiang Cancer Hospital, 1 East Banshan Road, Hangzhou 310022, China

E-mail: chenming@zjcc.org.cn

（Professor Ming Chen has been worked in Department of Radiation Oncology, Sun Yat-sen University Cancer Center since 2022）

Professor Xianghui Du

Department of Radiation Oncology, Zhejiang Cancer Hospital, 1 East Banshan Road, Hangzhou 310022, China

E-mail: duxh@zjcc.org.cn

Index

Schema
Outline
1.0 Background
2.0 Objectives
3.0 Patient Selection
4.0 Pretreatment Evaluations
5.0 Randomization
6.0 Therapeutic protocols
7.0 Response evaluation
8.0 Toxicity assessment
9.0 Follow-up
10.0 Statistical analysis
Reference

Schema ↓

Esophageal Cancer

Staging: IB ~ ⅣB by AJCC 6th edition

Age: 70- 85 years

↓

REGISTRATION

↓

STRATIFICATION

(By age and tumor length)

↓

RANDOMIZATION

**Group 1:** Chemoradiotherapy with S-1	**Group 2:** Radiotherapy alone
Chemotherapy: S-1 70mg/m^2/day, twice per day, day1-14 and day 29-42; Radiation: 54 Gy, 1.8 Gy per fraction, 30 fractions, 6 weeks	Radiation: 60 Gy, 2 Gy per fraction, 30 fractions, 6 weeks

↓

Follow-up

Outline

Official title: Concurrent chemoradiotherapy with S-1 versus radiotherapy alone in elderly patients with esophageal cancer: a multicenter, randomized, phase III trial.
Study Type: Clinical trial proposed by physicians.
Participants: Elderly esophageal cancer patients.
Study Design: Randomized, phase 3, multicenter, open-label trial.
Estimated sample size: 298 participants.
Inclusion Criteria:

1. Patients with newly histologically or cytologically confirmed esophageal carcinoma.
2. Stage IB ~ ⅣB according to the 6th AJCC edition staging (including the stage IVB of supraclavicular or celiac lymph nodes metastasis, but excluding stage IVB of other distant metastasis).
3. Patients aged from 70 to 85 years old.
4. Eastern Cooperative Oncology Group (ECOG) performance score 0~1.
5. Have no history of esophageal perforation, active esophageal bleeding, and obvious invasion of trachea or thoracic large vessels.
6. Eligible patients had no history of previous anticancer therapy (including radiotherapy and chemotherapy).
7. Adequate marrow: hemoglobin ≥ 9 g/dL, WBC 3.0×10^9/L, neutrophils $\geq1.5\times10^9$/L, platelet $\geq100 \times 10^9$/L.
8. Adequate liver and kidney function: serum creatinine (Scr) ≤ 1.5 ULN, serum bilirubin ≤ 1.5 ULN， alanine aminotransferase (ALT) and aspartate aminotransferase (AST) ≤ 2.5 ULN (note: ULN = Upper limit of normal).
9. No history of interstitial pneumonia
10. FEV1 ≥ 0.8L.
11. Signed study-specific consent form prior to study entry.

Exclusion Criteria:

1. Patients with hematogenous metastases or distant lymph node metastases (other than metastasis to supraclavicular or celiac lymph nodes). Patients with multiple carcinomas of the esophagus. Patients with malignant pleural effusion or pericardial effusion.
2. A history of previous radiotherapy, chemotherapy, or surgery to the primary tumor or nodes.
3. Tracheoesophageal fistula, invasion of the primary tumor into the trachea or major bronchi, deep esophageal ulcer, or hematemesis.
4. Severe comorbidities such as active infection, cardiovascular or pulmonary disease.
5. Prior or concurrent malignancies excepting for adequately treated skin cancer (non-melanoma).
6. Patients participated in other clinical trials within 30 days.
7. Any other condition which in the investigator' s opinion should be excluded from this trial.

Study start date: June 2016.
Estimated completion date: June 2019.
Chemoradiotherapy group: combined therapy including radiation, 54 Gy, 1.8 Gy per fraction, 30 fractions, 6 weeks and S-1 70mg/m^2/day, oral administration, bid, once in the mornings and once at nights, day1-14 and day 29-42.
Radiotherapy group: radiation, 60 Gy, 2 Gy per fraction, 30 fractions, 6 weeks.

Primary Endpoint: 2-year overall survival.
Secondary endpoints: progression-free survival (PFS), response rates and toxicity profile.

1.0 Background

Esophageal cancer is a major public health problem in China. The estimated number of new esophageal cancer cases and deaths in 2011 were 291,238 and 218,957, respectively.[1,2] Esophageal cancer often occurs in elderly patients. Approximately 30-40% of esophageal cancer patients were 70 years old or older.[3] Because of the rapid aging population and increasing life expectancy, the number of elderly patients in China is likely to increase significantly in the future.

Surgery is the mainstay radical treatment for esophageal cancer. However, elderly patients were more likely to be referred to non-surgical treatment, partially because of medical comorbidities and reduced functional reserve of organs. [4–6] A population-based study of the National Cancer Registry in Ireland showed that, when compared with patients younger than 60 years of age, the likelihood for resection was significantly lower among older cohorts by 33%, 74% and 93% for patients aged 60–69, 70–79 and 80+, respectively.[6]

RTOG 85-01, a prospective randomized trial by Radiation Therapy Oncology Group, demonstrated that concurrent cisplatin and fluorouracil chemoradiotherapy (CCRT) provided a significant survival advantage over radiation alone.[7] CCRT is now the standard treatment for unresectable locoregionally advanced esophageal carcinoma. However, the incidence of severe and life-threatening side effects was higher in CCRT group than RT alone group. Only 23% of patients were 70 years or older in this study, no outcome and toxicity report was seen for this subgroup.[7,8] Recently, several studies reported that a selected population of elderly esophageal carcinoma patients could benefit from concurrent cisplatin and fluorouracil chemoradiotherapy, with clinical complete response (CR) rate of 57.8% to 63.6% and survival time of 8.6-15.2 months. However, only 9-38.5% of patients finished the protocol-defined treatment in these studies.[9–12] Moreover, it was reported that treatment-related death was suspected in up to 18%.[12] Therefore, it is of great importance to find a lower toxicity regimen for the elderly population.

S-1 is an orally administered compound preparation, which could enhance anticancer activity and reduce toxicity through the combined use of an oral fluoropyrimidine agent (tegafur), a dihydropyrimidine dehydrogenase inhibitor (5-chloro-2,4-dihydroxypyridine), and an orotate phosphoribosyl transferase inhibitor (potassium oxonate).[13] It has been confirmed that S-1 had excellent efficiency in metastatic stomach cancer, non-small cell lung cancer, and colorectal cancer with mild toxicity.[14–16] S-1 also has several advantages over 5-FU in CCRT. First, prolonged exposure is desirable in order to achieve radiosensitisation. Studies have shown that the half-life of 5-FU after oral S-1administration was markedly prolonged compared with that of 5-FU after intravenous administration.[13] Second, Gimeracil, a component of S-1, has been found to enhance the efficacy of RT through the inhibition of the repair of radiation-induced DNA damage.[17] A preclinical study using human cancer xenograft models showed that oral S-1 produced better response than intravenous 5-FU in the chemoradiotherapy.[18] Moreover, S-1 is administered orally, it can be given on a daily basis, which is convenient for fractionated radiotherapy.

For these reasons, we conducted a phase I trial of S-1 with concurrent radiotherapy in elderly esophageal cancer patients. This regimen was proved to be feasible and well tolerated in the study population. The median survival time was 29 months. Esophagitis was the most common toxicity; grade 3 esophagitis was observed in 3 of 12 patients (25%). No grade 4 toxicity or treatment-related death was observed in the study.[19] Subsequently, a phase II trial of S-1 with concurrent radiotherapy was conducted in elderly patients with esophageal cancer. From October 2012 to October 2015, 30 patients were enrolled. The grade 3 toxicities included esophagitis (16.7%), leucopoenia (13.3%), neutropenia (10%), anemia (3.3%), pneumonitis (3.3%) and fatigue (3.3%). No grade 4 toxicity or treatment-related death occurred. A total of 29 patients were eligible for response evaluation, complete response rate and partial response rate was

20.6% (6 in 29) and 37.9% (11 in 29), respectively. Meanwhile, 41.3% (12 in 29) patients experienced stable disease. One patient who discontinued radiotherapy at 28.8Gy did not receive response evaluation. Based on these results, we conducted a multi-center, randomized, phase III trial to further evaluate the effectiveness and safety of S-1 concurrent chemoradiotherapy versus radiotherapy alone in the elderly patients with esophageal cancer.

2.0 Objectives

To compare the efficacy and safety of concurrent chemoradiotherapy with S-1 versus radiotherapy alone in elderly patients with esophageal cancer.

The primary endpoint is 2-year overall survival (OS), which is calculated from date of randomization to death for any cause or the last follow-up.

The secondary endpoints include progression-free survival (PFS), treatment response, toxicity profile. PFS was calculated from date of randomization to disease progression or death. Treatment response will be evaluated according to the Response Evaluation Criteria in Solid Tumors Version 1.0 (RECIST v 1.0) guideline. Treatment associated toxicity will be evaluated according to the National Cancer Institute Common Terminology Criteria for Adverse Events Version 4.0 (CTCAE v 4.0).

3.0 Patient selection

3.1 Inclusion Criteria

1) Patients with newly histologically or cytologically confirmed esophageal carcinoma.
2) Stage IB ~ ⅣB according to the 6th AJCC edition staging (including the stage IVB of supraclavicular or celiac lymph nodes metastasis, but excluding stage IVB of other distant metastasis).
3) Patients aged from 70 to 85 years old.
4) Eastern Cooperative Oncology Group (ECOG) performance score 0~1.
5) Have no history of esophageal perforation, active esophageal bleeding, and obvious invasion of trachea or thoracic large vessels.
6) Eligible patients had no history of previous anticancer therapy (including radiotherapy and chemotherapy).
7) Adequate marrow: hemoglobin ≥ 9 g/dL, WBC 3.0×10^9/L, neutrophils $\geq1.5\times10^9$/L, platelet $\geq100\times10^9$/L.
8) Adequate liver and kidney function: serum creatinine (Scr) ≤ 1.5 ULN, serum bilirubin ≤ 1.5 ULN， alanine aminotransferase (ALT) and aspartate aminotransferase (AST) ≤ 2.5 ULN (note: ULN = Upper limit of normal).
9) No history of interstitial pneumonia
10) FEV1 ≥ 0.8L.
11) Signed study-specific consent form prior to study entry.

3.2 Exclusion Criteria

1) Patients with hematogenous metastases or distant lymph node metastases (other than metastasis to supraclavicular or celiac lymph nodes). Patients with multiple carcinomas of the esophagus. Patients with malignant pleural effusion or pericardial effusion.
2) A history of previous radiotherapy, chemotherapy, or surgery to the primary tumor or nodes.
3) Tracheoesophageal fistula, invasion of the primary tumor into the trachea or major bronchi, deep esophageal ulcer, or hematemesis.
4) Severe comorbidities such as active infection, cardiovascular or pulmonary disease.
5) Prior or concurrent malignancies excepting for adequately treated skin cancer (non-melanoma).

6) Patients participated in other clinical trials within 30 days.
7) Any other condition which in the investigator' s opinion should be excluded from this trial.

4.0 Pretreatment evaluations

All patients should receive the following examinations before recruitment within 14 days before treatments.

1) Complete history and physical examination, including the patient's weight, assessments of the patient's performance status, nutritional risk screening 2002, Charlson comorbidity index.
2) Laboratory Studies: CBC, ANC, platelets; Blood biochemistry include liver and kidney function, blood electrolytes.
3) Electrocardiography.
4) Lung function tests.
5) Histopathology. Biopsy of supraclavicular node if clinically enlarged.
6) Imaging Studies: neck-thorax-abdomen CT; Upper GI endoscopy, endoscopic ultrasound; If necessary, ECT and brain MRI will be employed to exclude metastasis; PET-CT scan (optional).
7) If necessary, electronic bronchoscopy will be employed to confirm the involvement of trachea and/or bronchus.

5.0 Randomization

Eligible patients are randomly assigned (1:1) to receive either chemoradiotherapy or radiotherapy alone. Randomization is stratified according to age (< 80 years vs. ≥ 80 years) and tumor length (< 5 cm vs. ≥ 5 cm). Block randomization will be carried out by a statistician, who is independent of the trial. Each block contains the patients in equal proportion. Patients and treating physicians are not masked.

6.0 Therapeutic protocols

6.1 Chemoradiotherapy group

6.1.1 Chemotherapy

1) Patients will receive two cycles of S-1 starting with radiotherapy: S-1 70 $mg/m^2/d$, bid, once in the mornings and once at nights, orally administered 30 min after meals, from day 1 to 14 and day 29 to 42 (Table 1). Because S-1 is only available for use in 20mg capsules in our trial, the individual dose was rounded down to the nearest pill size less than the calculated dose. A powder form of S-1 would be administered if patients could not swallow the oral capsule.

Table 1. Time schedule of concurrent chemo-radiotherapy

Radiotherapy 6 weeks		
S-1: day 1 to 14	Intermission	S-1: day 29 to 42

2) Dose modifications

Chemotherapy should be withheld in the conditions as follows: ANC < 1.5 $\times 10^9$/L; Plt < 75 $\times 10^9$/L; ≥ Grade 3 nonhematological toxicities. Up to a 2-week delay of chemotherapy is allowed, or the chemotherapy will be terminated. S-1 dose will be reduced by 25% if the patient has an episode of ANC < 0.5 $\times 10^9$/L, or Plt < 50 $\times 10^9$/L. Two times of dose reduction is allowed for S-1, otherwise chemotherapy will be terminated if the patient has third episode of the mentioned toxicity.

6.1.2 Radiation

Radiation will be delivered by three-dimensional conformal radiotherapy or intensity-modulated radiotherapy (IMRT) using 6-10 MV photons.

1) Fixation and simulation

Patients are immobilized in the supine position with a thermoplastic mask or a vacuum bag. A CT-sim scan with 5 mm slice thickness and intravenous contrast will be performed in the treatment position including neck, thorax and abdomen.

2) Treatment plan

Window width of 400 Hu and window level of +20 Hu is recommended to use in delineating primary tumor and positive lymph node.

a. Gross tumor volume (GTV): GTV includes the primary tumor (GTV-T) and the enlarged lymph nodes (GTV-N), which will be determined using all available information (physical examination, endoscopy, EUS, neck-thorax-abdomen CT, PET-CT, etc.).

Clinic tumor volume (CTV): The clinical tumor volume (CTV) is defined as the primary tumor plus 3 cm expansion superiorly and inferiorly, and 1cm radial expansion. The nodal CTV is defined as the metastatic lymph node plus 0.5-1.0 cm expansion. Elective radiation including supraclavicular lymph nodes region for primary tumors of the upper third of the esophagus and the coeliac lymph nodes region for primary tumors of the lower third of the esophagus will be conducted in patients aged 70-79 years old. Patients with 80 years or older dispense with prophylactic radiation for elective neck area.

Plan tumor volume (PTV): PTV is designed for tumor motion and set-up variations. If 4D-CT SIM is used, PTV is defined as CTV plus a 0.5 cm margin. If 3D-CT SIM is used, PTV is defined as CTV plus a 0.5-1 cm margin.

3）Radiation dose

The prescription dose is 54 Gy, 1.8 Gy per fraction, 30 fractions, 6 weeks. 95% prescription dose should cover plan target volume. Consecutive volume with dose ≥120% of the prescribed dose in PTV should be less than 2 cm^3, consecutive volume with dose ≥110% of the prescribed dose out of PTV should be less than 1 cm^3.

4）Organs at risk (OARs) include both lung, heart, spinal cord, stomach and liver. Dose-Volume-Histograms (DVHs) will be used to select the most appropriate treatment plan and to evaluate the damage risk of normal tissue. Standard dose constraints are applied for treatment plan: a) Spinal cord: maximum dose < 45 Gy. b) Lungs: mean dose (bilateral) <15 Gy, total volume receiving greater than 20 Gy (V20) < 30%, every effort should be made to keep the total lung dose to a minimum. c) Heart: mean dose < 30 Gy. d) Liver: mean dose < 25 Gy. e) Stomach: maximum dose < 54 Gy, total volume receiving greater than 40 Gy (V40) < 40%.

5) Radiation modifications

If the patient develops ≥ grade 3 RT-related toxicity, radiotherapy should be withheld. Treatment can resume once grade 3 RT-related toxicity is no longer present. It's better to resume radiation within 5 days. Radiotherapy suspension is no longer than 2 weeks, otherwise it would be terminated. Active symptomatic treatment, like antibiotics combined with corticosteroid and/or a feeding tube for ≥ grade 3 esophagitis, G-CSF for ≥ grade 3 neutropenia, etc, could be administered. If any of the following situation is present, patients will be excluded from the treatment protocol: esophageal perforation and heavy hemorrhage, non-healing esophageal tracheal leakage, myocardial infarction, heart failure, severe arrhythmias, radiation pneumonia with dyspnea, and treatment interruption ≥ 2 weeks.

6.2 Radiotherapy group

Radiation dose

The prescription dose is 60 Gy, 2 Gy per fraction, 30 fractions, 6 weeks.

Fixation, simulation, treatment plan, standard dose constraints of OARs and radiation modification is the same with chemoradiotherapy group.

7.0 Response evaluation

Treatment response is evaluated 4 weeks after completion of radiation according to RECIST 1.0 standard. Patients will receive workup including EGD, esophageal barium, and neck, chest and abdominal CT (plain and enhanced) from the first cervical spine to the umbilical plane.

8.0 Toxicity assessment

Toxicity is assessed and graded according to the National Cancer Institute Common Terminology Criteria for Adverse Events v4.0 (CTCAE v4.0).

9.0 Follow-up

After completion of the treatment, patients will be re-examined every 3 months during the first 2 years, and every 6 months thereafter. Physical examination, ECG, Esophageal barium, and neck/chest/abdominal CT with contrast will be performed at follow-up visits. Gastroscopy would be performed at first visit. The ECOG score and survival status are recorded at each visit. Whenever possible, locoregional or distant recurrences are confirmed by fine needle aspiration or biopsy. Clinical diagnosis is accepted for sites where biopsy tissue could not be obtained but present with classic changes (with or without clinical symptoms) on at least two imaging methods, including PET-CT, MRI, CT, bone scans, and abdominal sonography. However, if imaging findings are equivocal, subsequent follow-up (eg. disease progression) would be used to ascertain the diagnosis. Each of the endpoints is assessed by the physician-in-charge. Patients who withdraw from this treatment protocol will also be followed up, and included for statistical analysis. When a participant death is reported, the researchers will manually fill in the death case report form.

10.0 Statistical analysis

Base on the RTOG 8501 and the previous results of similar patients, the 2-year overall survival rate in the radiotherapy group was assumed to be 16%. We calculated that a total enrollment of 298 patients was needed to detect an improvement of 14% in OS at 2 years (30% in chemoradiotherapy group) via a log-rank test with two-sided alpha of 0.05, and a statistical power of 90%, assuming 3 years of patient accrual, 2 years of follow-up and 10% patient loss.

Efficacy analyses will be done in the intention-to-treat population. Overall survival (OS) is defined as the time period from the date of randomization to the date of death or the last follow-up. OS including 1-year, 2-year and 3-year survival rates will be calculated using the Kaplan-Meier method, and then compared by the log-rank test. PFS is defined as the time period from date of randomization to disease progression or death. PFS will be calculated using the Kaplan-Meier method, and then compared by the log-rank test. The treatment response will be evaluated according to the Response Evaluation Criteria in Solid Tumors Version 1.0 (RECIST v 1.0) guideline. Treatment associated toxicity will be evaluated according to the National Cancer Institute Common Terminology Criteria for Adverse Events Version 4.0 (CTCAE v 4.0). Pearson's x^2 or Fisher's exact test was used to compare response rates, toxicity rates, and other categorical variables between the two groups. P values of less than 0.05 were considered to indicate statistical significance.

Reference

1. Chen W, Zheng R, Baade PD, et al. Cancer statistics in China, 2015. *CA Cancer J Clin*. 2016;66(2):115-132.
2. Zeng H, Zheng R, Zhang S, et al. Esophageal cancer statistics in China, 2011: Estimates based on 177 cancer registries. *Thorac Cancer*. 2016;7(2):232-237.

3. Wu M, van't Veer P, Zhang ZF, et al. A large proportion of esophageal cancer cases and the incidence difference between regions are attributable to lifestyle risk factors in China. *Cancer Lett*. 2011;308(2):189-196.
4. Steyerberg EW, Neville B, Weeks JC, Earle CC. Referral patterns, treatment choices, and outcomes in locoregional esophageal cancer: A population-based analysis of elderly patients. *J Clin Oncol*. 2007;25 (17):2389-2396.
5. Tougeron D , Hamidou H , Michel Scotté, et al. Esophageal cancer in the elderly: an analysis of the factors associated with treatment decisions and outcomes. BMC Cancer. 2010;10(1):1-10.
6. Cronin-Fenton DP, Sharp L, Carsin AE, Comber H. Patterns of care and effects on mortality for cancers of the oesophagus and gastric cardia: A population-based study. *Eur J Cancer*. 2007;43 (3):565-575.
7. Herskovic A, Martz K, al-Sarraf M, et al. Combined chemotherapy and radiotherapy compared with radiotherapy alone in patients with cancer of the esophagus. *N Engl J Med*. 1992;326(24):1593-1598.
8. Cooper JS, Guo MD, Herskovic A, et al. Chemoradiotherapy of locally advanced esophageal cancer: long-term follow-up of a prospective randomized trial (RTOG 85-01). Radiation Therapy Oncology Group. *JAMA*. 1999;281(17):1623-1627.
9. Tougeron D, Di Fiore F, Thureau S, et al. Safety and outcome of definitive chemoradiotherapy in elderly patients with oesophageal cancer. *Br J Cancer*. 2008;99(10):1586-1592.
10. Takeuchi S, Ohtsu A, Doi T, et al. A retrospective study of definitive chemoradiotherapy for elderly patients with esophageal cancer. *Am J Clin Oncol*. 2007;30(6):607-611.
11. Semrau R, Herzog SL, Vallbohmer D, Kocher M, Holscher A, Muller RP. Radiotherapy in elderly patients with inoperable esophageal cancer. Is there a benefit? *Strahlenther Onkol*. 2012;188(3):226-232.
12. Wakui R, Yamashita H, Okuma K, et al. Esophageal cancer: definitive chemoradiotherapy for elderly patients. *Dis Esophagus*. 2010;23(7):572-579.
13. van Groeningen CJ, Peters GJ, Schornagel JH, et al. Phase I clinical and pharmacokinetic study of oral S-1 in patients with advanced solid tumors. *J Clin Oncol*. 2000;18(14):2772-2779.
14. Boku N, Yamamoto S, Fukuda H, et al. Fluorouracil versus combination of irinotecan plus cisplatin versus S-1 in metastatic gastric cancer: a randomised phase 3 study. *Lancet Oncol*. 2009;10(11):1063-1069.
15. Sakuramoto S, Sasako M, Yamaguchi T, et al. Adjuvant chemotherapy for gastric cancer with S-1, an oral fluoropyrimidine. *N Engl J Med*. 2007;357(18):1810-1820.
16. Van Den Brande J, Schöffski P, Schellens JHM, et al. EORTC Early Clinical Studies Group early phase II trial of S-I in patients with advanced or metastatic colorectal cancer. *Br J Cancer*. 2003;88(5):648-653.
17. Takagi M, Sakata K, Someya M, et al. Gimeracil sensitizes cells to radiation via inhibition of homologous recombination. *Radiother Oncol*. 2010;96(2):259-266.
18. Fukushima M, Sakamoto K, Sakata M, Nakagawa F, Saito H, Sakata Y. Gimeracil, a component of S-1, may enhance the antitumor activity of X-ray irradiation in human cancer xenograft models in vivo. *Oncol Rep*. 2010;24(5):1307-1313.
19. Ji Y, Qiu G, Sheng L, et al. A phase I dose escalation study of S-1 with concurrent radiotherapy in elderly patients with esophageal cancer. *J Thorac Dis*. 2016;8(3):451-458.
