# Supplementary material for: Long-Term Outcomes of Concurrent Chemoradiotherapy With S-1 in Older Patients With Esophageal Cancer: A Secondary Analysis of a Randomized Clinical Trial
Source: JAMA Netw Open. 2026 Mar 27;9(3):e263541. doi: 10.1001/jamanetworkopen.2026.3541 (PMC13032157; doi:10.1001/jamanetworkopen.2026.3541)
Supplement: Supplement 2. — eTable 1. Overall Survival and Progression-Free Survival in 298 Patients: Multivariate Cox Proportional Analysis eTable 2. All Causes of Death Recorded Until the Final Day of Follow-up eTable 3. Site of First Progression eFigure 1. Restricted Mean Survival Time eFigure 2. Subgroup Analyses of Overall Survival in the Intention-to-Treat Population eFigure 3. Cancer-Specific Survival [file jamanetwopen-e263541-s002.pdf]

Supplemental Online Content

Ji Y, Fang M, Zhu W, et al. Long-term outcomes of concurrent chemoradiotherapy with S-1 in older patients with esophageal cancer: a secondary analysis of a randomized clinical trial. *JAMA Netw Open*. 2026;9(3):e263541. doi:10.1001/jamanetworkopen.2026.3541

eTable 1. Overall Survival and Progression-Free Survival in 298 Patients: Multivariate Cox Proportional Analysis
eTable 2. All Causes of Death Recorded Until the Final Day of Follow-up
eTable 3. Site of First Progression
eFigure 1. Restricted Mean Survival Time
eFigure 2. Subgroup Analyses of Overall Survival in the Intention-to-Treat Population
eFigure 3. Cancer-Specific Survival

This supplemental material has been provided by the authors to give readers additional information about their work.

© 2026 Ji Y et al. *JAMA Network Open*

eTable 1. Overall Survival and Progression-Free Survival in 298 Patients: Multivariate Cox Proportional Analysis

Variables	Comparison	OS		PFS	
		HR (95% CI)	P Value	HR (95% CI)	P Value
Treatment arm	RT vs CCRT	0.68 (0.52-0.88)	0.004	0.69 (0.53-0.90)	0.006
Age	70-79 years vs ≥80 years	1.44 (1.08-1.92)	0.014	1.33 (1.00-1.78)	0.050
Sex	Female vs Male	1.38 (1.05-1.80)	0.021	1.30 (1.00-1.69)	0.052
Charlson Comorbidity Index	0 vs ≥1	1.34 (1.00-1.80)	0.050	1.27 (0.96-1.66)	0.090
Stage (AJCC, 6th edition)	II vs III-IV	1.33 (1.01-1.75)	0.042	1.27 (0.97-1.67)	0.082

Abbreviations: HR, hazard ratio; CCRT, concurrent chemoradiotherapy; RT, radiotherapy.

Hazard Ratio: a hazard ratio of 1 indicates no difference between the 2 subgroups. The variables were coded such that a HR greater than 1 indicates an increased risk of death or progression for the second level of the variables listed.

© 2026 Ji Y et al. *JAMA Network Open*

eTable 2. All causes of death recorded until the final day of follow-up

Cause of death	CCRT (N=149)	RT (N=149)
Death of EC	86	105
Death of other causes	17	15
Treatment related	3	4
Cardiovascular	3	3
Pulmonary	5	2
Covid-19	2	2
Other	4	4
Death of unknown	4	2

© 2026 Ji Y et al. *JAMA Network Open*

eTable 3. Site of first progression

	CCRT (n=149)	RT (n=149)	$\chi 2$	P
All cases with progression	91 (61.1)	107 (71.8)	3.386	0.066
Locoregional only	48 (32.2)	70 (47.0)	6.187	0.013
Distant only	29 (19.5)	22 (14.8)	0.852	0.356
Locoregional and distant	14 (9.4)	15 (10.1)	0	1.000

Abbreviations: CCRT, concurrent chemoradiotherapy; RT, radiotherapy.

Data are presented as number (%).

© 2026 Ji Y et al. *JAMA Network Open*

eFigure 1. Restricted mean survival time.

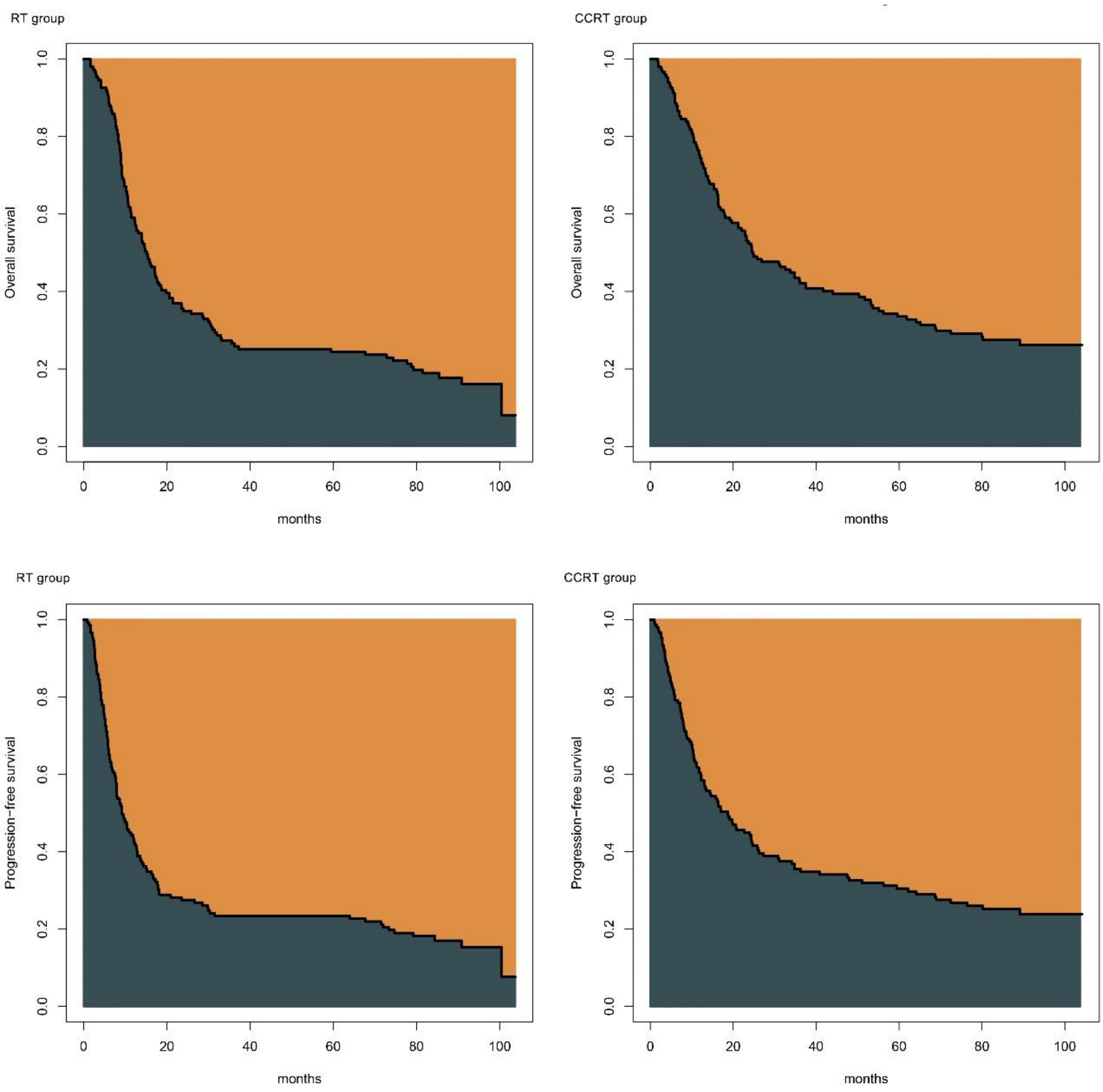

eFigure 1. Restricted mean survival time of overall survival and progression-free survival.

© 2026 Ji Y et al. *JAMA Network Open*

eFigure 2. Subgroup analyses of overall survival in the intention-to-treat population.

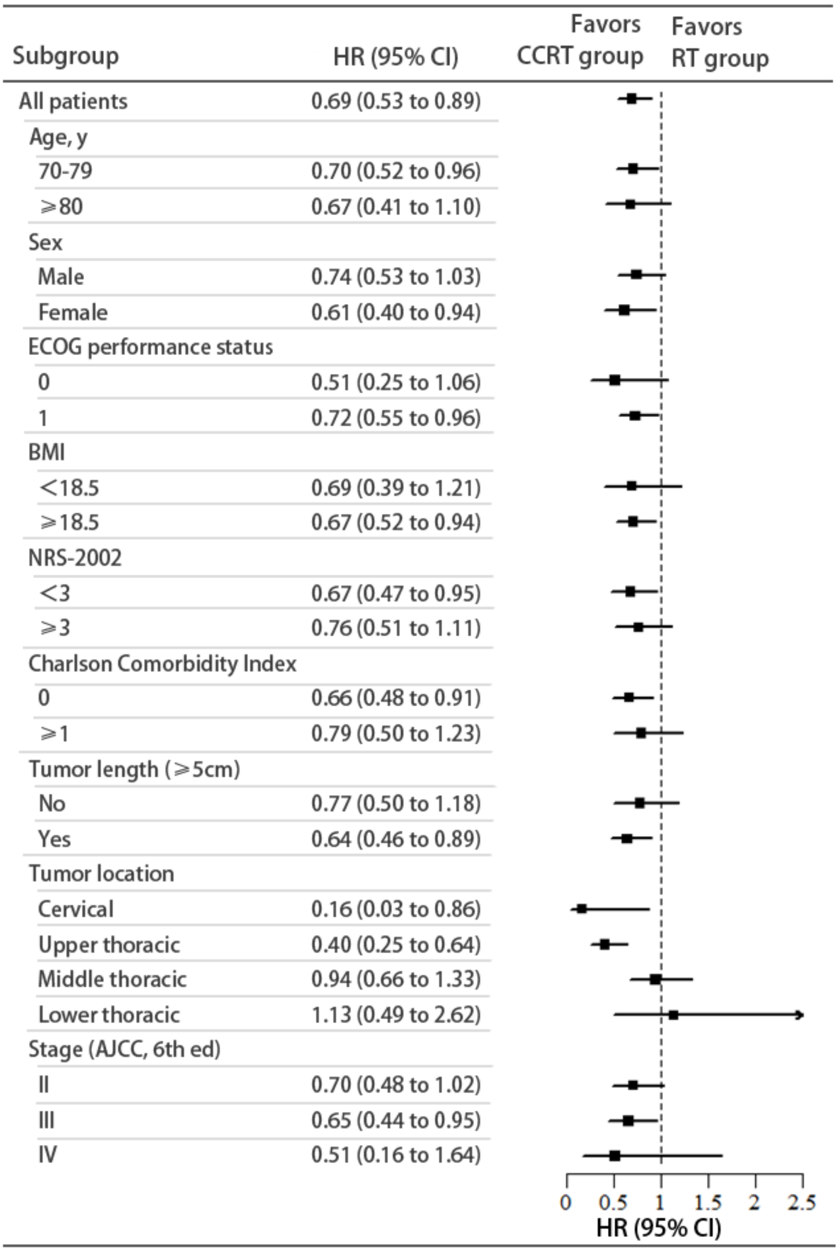

eFigure 2. Subgroup analyses of overall survival in the intention-to-treat population. CCRT, concurrent chemoradiotherapy; RT, radiotherapy; ECOG, Eastern Cooperative Oncology Group; BMI, body mass index; NRS-2002, nutritional risk screening 2002; AJCC, American Joint Committee on Cancer.

© 2026 Ji Y et al. *JAMA Network Open*

eFigure 3. Cancer-specific survival.

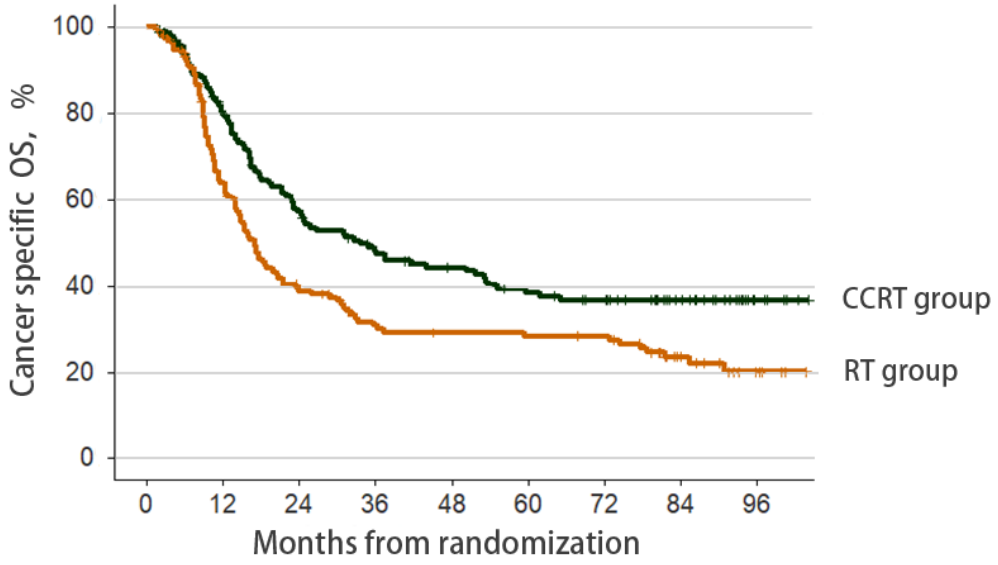

No. at risk

CCRT group	149	112	78	62	54	46	41	27	6
RT group	149	88	52	37	34	33	32	16	5

eFigure 3. Cancer-specific survival in CCRT and RT group (HR, 0.64; 95% CI, 0.48-0.85; *P* = .002). CCRT, concurrent chemoradiotherapy; RT, radiotherapy.

© 2026 Ji Y et al. *JAMA Network Open*
