# Supplementary material for: Long-Term Outcomes of Concurrent Chemoradiotherapy With S-1 in Older Patients With Esophageal Cancer: A Secondary Analysis of a Randomized Clinical Trial
Source: JAMA Netw Open. 2026 Mar 27;9(3):e263541. doi: 10.1001/jamanetworkopen.2026.3541 (PMC13032157; doi:10.1001/jamanetworkopen.2026.3541)
Supplement: Supplement 3. — Data Sharing Statement [file jamanetwopen-e263541-s003.pdf]

Data Sharing Statement

Ji. Long-Term Outcomes of Concurrent Chemoradiotherapy With S-1 in Older Patients With Esophageal Cancer. *JAMA Netw Open*. Published March 27, 2026.
doi:10.1001/jamanetworkopen.2026.3541

Data
Additional Information: S-1 Concurrent With Radiotherapy Versus Radiotherapy for Elderly Patients With Esophageal Cancer, https://clinicaltrials.gov/, NCT02813967
Data available: No

Additional Information
Explanation for why data not available: The datasets used in the current study are available from the corresponding author on reasonable request.
